# Genome-wide identification and comparative analysis of strigolactone biosynthetic genes in major solanaceous crops

**DOI:** 10.1371/journal.pone.0338033

**Published:** 2025-12-04

**Authors:** Ranbo Guo, Xin Li, Can Zhu, Lei Liu, Chunyang Pan, Junling Hu, Xiaoxiao Lu, Shumin He, Lin Yang, Yanmei Guo, Zejun Huang, Xiaoxuan Wang, Yongchen Du, Wenying Zhu, Junming Li

**Affiliations:** 1 College of Horticulture, Qingdao Agricultural University, Qingdao, China; 2 State Key Laboratory of Vegetable Biobreeding, Institute of Vegetables and Flowers, Chinese Academy of Agricultural Sciences, Beijing, China; Graphic Era Institute of Technology: Graphic Era Deemed to be University, INDIA

## Abstract

Strigolactones (SLs) are a class of important plant hormones that not only regulate plant growth and development but also mediate responses to biotic and abiotic stresses. Functioning as signaling molecules derived from the rhizosphere, SLs play a pivotal role in inducing the germination and facilitating the parasitism of root holoparasites such as *Orobanche* spp. Notably, the parasitism success of *Orobanche* exhibits marked variation within Solanaceous crops, demonstrating dual dependence on SL exudation dynamics and edaphic phosphate bioavailability. We performed a genome-wide characterization of SL biosynthetic orthologs across nine Solanaceous species through integrated phylogenomic pipelines alongside qRT-PCR expression profiling. In this study, we identified 113 putative SL biosynthetic orthologs across wolfberry, nightshade, tomato, eggplant, petunia, tobacco, pepper, groundcherry and potato, revealing both deep evolutionary conservation and lineage-specific diversification patterns. Phosphate deprivation significantly induced the upregulation of SL biosynthetic genes in tomato and pepper via qRT-PCR analysis, confirming that phosphorus deficiency acts as a key stimulator of SL biosynthesis. In general, this study delineates the repertoire of potential SL biosynthesis-related genes across major Solanaceous species, revealing phylogenetic conservation and clade-specific diversification.

## Introduction

Strigolactones (SLs) are a class of phytohormones derived from terpenoids that regulate various plant developmental processes, including shoot branching, tissue regeneration, and root system development [1]. They also play a key role in plant responses to biotic and abiotic stresses [2]. Apart from their endogenous roles, SLs exuded from roots serve as rhizospheric signals, inducing the germination of parasitic plant seeds and enhancing symbiotic associations with soil microorganisms [3–5]. Previous studies have shown that SLs are structurally diverse and their biosynthetic network is correspondingly complex [6]. The biosynthesis begins with all-trans-β-carotene, which is transformed into carlactone (CL) through a series of enzymatic reactions mediated by D27, CCD7, and CCD8, and is then oxidized by CYP711A to form carlactonoic acid (CLA) [6]. This core pathway is highly conserved across different plant species. However, the downstream conversion of CLA into distinct SLs is strongly species-specific and is predominantly shaped by oxidative modifications mediated by cytochrome P450 (CYP450) enzymes [6]. SLs are categorized based on structural characteristics into canonical and non-canonical types. In the canonical pathway, CYP722C2 mediates the synthesis of 4-deoxyorobanchol in rice, while CYP722C and CYP712G are responsible for orobanchol and solanacol production in tomato [7,8]. In sorghum, CYP728B contributes to sorgomol formation [9]. In contrast, non-canonical SLs such as 1’-OH-MeCLA and zealactone, which is produced in maize, are synthesized through the coordinated action of CYP706C, CLAMT, and LBO [10,11]. Currently, the biosynthetic pathways and functional roles of both SL types remain incompletely understood, and interspecies differences in SL biosynthetic pathways require further investigation.

Parasitic weeds of *Orobanche* spp. have posed a serious threat to global crop production, with infestations that span up to 60 million hectares annually and result in multibillion-dollar economic losses worldwide [12]. These obligate holoparasites detect host root-secreted SLs to initiate seed germination and subsequently recognize additional host signals to establish parasitism via other signaling pathways [13,14]. Upon successful attachment, the parasite extracts water and nutrients from the host, resulting in substantial crop yield losses [15,16]. Recent studies indicate that soil nutrient status, particularly phosphate (Pi) availability, critically modulates the parasitism efficacy of *Orobanche*. Pi deficiency markedly induces SL biosynthesis genes in host plants, elevating rhizosphere SL levels and promoting *Orobanche* infestation [17,18].

Beyond abiotic factors, host species critically govern parasitism severity. Within the Solanaceae family, resistance levels vary substantially across species, yet the mechanistic basis for this variation remains poorly understood [19]. For instance, tomato and tobacco, two widely cultivated Solanaceous crops, are generally highly susceptible to *Orobanche*, and resistance breeding has been hindered by limited genetic resistance resources [20]. Meanwhile, rapid physiologic race differentiation in the parasite impedes durable host resistance development [21]. In contrast, *Orobanche* infection is seldom reported in pepper and eggplant. Although pepper roots induce higher *Orobanche* germination rate than tomato under equivalent conditions, parasitism incidence remains substantially lower, with sweet pepper exhibiting near-complete resistance [22]. Advancing *Solanaceae* genomic datasets have enabled the identification of significant synteny across different species [23]. However, a systematic evolutionary framework for SL biosynthetic genes in Solanaceous crops has not yet been established. While previous efforts have systematically identified SL-related genes in several model species, including Arabidopsis, rice, maize, and tomato, these studies have been largely confined to individual species. A systematic, comparative analysis across multiple species within a single plant family is still lacking. Such a comparative approach is crucial to move beyond cataloging genes in isolated systems and to uncover the evolutionary dynamics—such as gene family expansion, contraction, and positive selection—that have shaped the diversity of SL pathways and may underlie the observed interspecific differences in *Orobanche* resistance.

The intersection of species-specific SL biosynthesis and the observed variation in Obanche resistance within the Solanaceae presents a compelling evolutionary puzzle. We posited that if the diversification of SL pathways constitutes a key determinant of host susceptibility, this should be reflected in distinct evolutionary signatures between susceptible and resistant species. To investigate this premise, we performed a comparative genomic and evolutionary analysis of SL biosynthetic genes across nine solanaceous species, establishing a systematic phylogenetic framework for these genes within major Solanaceae lineages. Specifically, we aimed to: (1) systematically identify and characterize the core components of the SL biosynthetic pathway; (2) compare evolutionary patterns—including gene family expansion and positive selection—between susceptible (e.g., tomato) and resistant (e.g., pepper) lineages; and (3) analyze the transcriptional dynamics of these genes under phosphate-deficient conditions to elucidate potential regulatory differences. These findings not only offer a theoretical foundation for elucidating SL pathway evolution in Solanaceae but also identify actionable targets for parasitic weed management.

## Materials and methods

### Genome-wide identification of SL biosynthetic genes

Genomic datasets used in this study were retrieved from publicly available genomic databases. Genome sequences of wolfberry (Lycium barbarum, PRJNA640228) and nightshade (Solanum americanum, PRJNA845062) were downloaded from NCBI database (https://www.ncbi.nlm.nih.gov/); those of tomato (Solanum lycopersicum, ITAG_4.0), eggplant (Solanum melongena, SME_HQ_1315), petunia (Petunia hybrida, P.axillaris_v.1.6.2), tobacco (Nicotiana tabacum, N.tabacum_v4.5), and pepper (Capsicum annuum, C.annuum_zunla_v2.0) were obtained from the SGN website (https://solgenomics.net/); groundcherry (Physalis peruviana, PRJCA002993) and potato (Solanum tuberosum, PRJCA011810) sequences were accessed from the NGDC database (https://ngdc.cncb.ac.cn/?lang=zh).

SL biosynthesis-related protein sequences were compiled from previous reports S1 Table, including D27 [24], CCD7 [25], CCD8 [26], CYP711A [27], CYP722C [7], and CYP712G [8] from tomato; LBO [28] and CLAMT [29] from Arabidopsis; CYP728B [9] from sorghum; and CYP706C [30] from rice. Homologous protein sequences were identified using BLASTP searches against the protein datasets of the nine Solanaceous species. The search parameters were optimized for each gene family based on sequence conservation. For the D27 family, a threshold of >30% identity and an e-value < 1e-5 was applied. For all other gene families (CCD7, CCD8, CYP711A, CYP722C, CYP712G, LBO, CLAMT, CYP728B, CYP706C), a more stringent threshold of >50% identity and an e-value = 0 was used. This differential strategy was employed because D27 constitutes a distinct gene family requiring broader criteria to capture divergent homologs, whereas the other genes belong to subfamilies within larger gene families (e.g., CYP450s) where stricter thresholds are necessary to ensure precise ortholog identification and avoid cross-annotation between closely related paralogs.

Conserved domain searches were conducted using hmmsearch based on Pfam domain models from the InterPro database (https://www.ebi.ac.uk/interpro/) [31]. Candidx`ate proteins were screened for characteristic domains, including D27-like_C (PF13225), RPE65 (PF03005), P450 (PF00067), Methyltransf_7 (PF03492), DIOX_N (PF14226), and 2OG-FeII_Oxy (PF03171). Gene structures were graphically represented using Chiplot website (https://www.chiplot.online/#) [32].

### Phylogenetic and promoter cis-element analyses

Full-length protein sequences of SL biosynthetic genes were aligned using MUSCLE [33]. Phylogenetic relationships were inferred using the Neighbor-Joining algorithm with the JTT model in TreeBest (https://treesoft.sourceforge.net/treebest.shtml), and branch support was evaluated with 1,000 bootstrap replicates.

Upstream 2-kb promoter regions of transcription start site were extracted and analyzed for cis-elements using the PlantCARE database (https://bioinformatics.psb.ugent.be/webtools/plantcare/html/) [34]. Element distributions were visualized using Chiplot website (https://www.chiplot.online/#) [32].

### Protein characteristics and subcellular localization

ProtParam website (https://web.expasy.org/protparam/) was used to analyze protein physicochemical properties, including molecular weight, isoelectric point, instability index, and hydrophilicity [35]. WoLF PSORT (https://www.genscript.com/wolf-psort.html) was used to predict subcellular localization. 3D protein structures were modeled using AlphaFold Server structure prediction platform (https://alphafoldserver.com/) [36].

### Selection pressure analysis

Homologous gene pairs between tomato and petunia, and between pepper and petunia were aligned using MUSCLE [33]. Non-synonymous substitution rates (Ka), synonymous substitution rates (Ks), and their ratios (Ka/Ks) were calculated using KaKs_Calculator 2.0 to evaluate evolutionary selection pressure [37].

### Pi stress treatments and sampling

Tomato cultivar ‘M82’ and pepper cultivar ‘Zunla-1’ were grown in seedling substrate (perlite: charcoal: vermiculite = 1:2:1), then the roots were rinsed at the four-leaf period of the seedlings and transferred to hydroponic culture with either Pi-deficient (Hoagland Modified Nutrient Salts Solution (-P), Coolaber, Cat# NS1010-P) or normal Hoagland solution (Hoagland Modified Nutrient Salts Solution, Coolaber, Cat# NSP1020). 16 h of light and 8 h of darkness were used to cultivate the seedlings at a constant temperature of 25°C for 1 week. Root samples were collected at the four-leaf stage (n = 3 biological replicates, each biological replicate consisted of 5 individual plants).

### Gene expression analysis

Total RNA was extracted from root tissues using a commercial RNA extraction kit (Huayueyang) following the manufacturer’s instructions.

First-strand cDNA was synthesized using the Hifair® II cDNA SuperMix (Yeasen), and qRT-PCR was performed using ChamQ SYBR Master Mix (Vazyme) on a Roche LightCycler 480 S2 Table.

## Results

### Identification and evolutionary analysis of SL biosynthetic genes

In this study, we systematically identified 10 gene families in the SL biosynthesis pathway and obtained 113 genes across nine Solanaceous species Table 1. Among these genes *Nicotiana tabacum* exhibited the largest set (19), while *Petunia hybrida* harbored the smallest set, with only 9 identified. Multiple *Solanaceae* species *(Solanum lycopersicum*, *Solanum melongena*, *Physalis peruviana*, *Capsicum annuum*, *Solanum tuberosum*, *Lycium barbarum*, and *Solanum nigrum)* also contained varying numbers of these genes. While the six core upstream genes (D27, CCD7, CCD8, CYP711A, CYP722C, and LBO) were universally conserved, we uncovered pronounced species-specificity in the downstream CYP450 complement. A key finding was the complete absence of the CLAMT gene across all nine species, despite the universal presence of LBO and CYP706C. Furthermore, we identified notable, lineage-specific expansion events, including tandem duplications of CYP706C in tomato, eggplant, and Physalis.

**Table 1:** The Number of SL Biosynthetic Genes in Different Species.

Species	D27	CCD7	CCD8	CYP711A	CYP722C	CYP712G	CYP728B	CYP706C	LBO	SUM
*Capsicum annuum* *(C.annuum_zunla_v2.0)*	4	1	1	1	1	0	1	2	1	12
*Solanum tuberosum* *(PRJCA011810)*	3	1	1	1	1	0	2	1	1	11
*Nicotiana tabacum* *(N.tabacum_v4.5)*	5	2	2	1	2	1	2	2	2	19
*Petunia hybrida* *(P.axillaris_v.1.6.2)*	3	1	1	1	1	0	1	0	1	9
*Physalis peruviana* *(PRJCA002993)*	1	1	1	1	1	1	1	5	1	13
*Solanum melongena* *(SME_HQ_1315)*	3	1	1	1	1	1	1	3	1	13
*Solanum lycopersicum* *(ITAG_4.0)*	3	1	1	1	1	1	1	6	1	16
*Solanum americanum* *(PRJNA845062)*	2	1	1	1	1	1	1	1	1	10
*Lycium barbarum* (PRJNA640228)	3	1	1	1	1	1	0	1	1	10

Note: Gene counts were derived from a systematic in silico analysis of the annotated genomes of nine Solanaceous species. The identification pipeline involved BLASTP and HMM searches with strict thresholds followed by manual validation for conserved domains (For the D27 family, a threshold of >30% identity and an e-value < 1e-5 was applied and a more stringent threshold of >50% identity and an e-value = 0 was used for all others). The counts are presented as the final number of putative genes identified per family per species using this standardized workflow.

**Fig 1:**
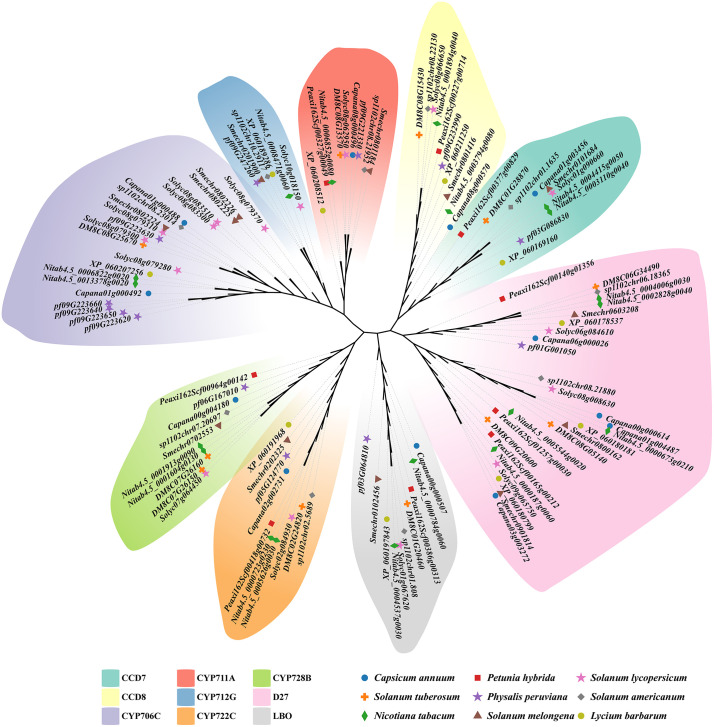
Phylogenetic tree of SL biosynthetic proteins from nine Solanaceous species. Proteins are color-coded by gene family and labeled by species, as indicated in the legend.

Gene identification was validated by constructing a phylogenetic tree based on full-length protein sequences of SL biosynthetic genes Fig 1. Most homologous sequences from different species clustered into highly conserved clades, reflecting close sequence homology and evolutionary conservation. Overall, the number and composition of SL biosynthetic genes varied significantly among species, suggesting that these genes have undergone varying degrees of conservation and diversification during evolution. These differences may be associated with species-specific functional requirements or environmental adaptations.

### Physicochemical characterization of SL biosynthetic proteins

A physicochemical analysis of the 113 SL biosynthesis-related proteins S2 and S3 Table revealed that the most of CYP450 family members consist of 392–572 amino acids. However, four CYP706C proteins from eggplant, Physalis, tobacco, and potato, together with one CYP728B protein from tobacco, exhibited notably longer sequences than others, suggesting the possible presence of expanded domains or specialized functional modules. Hydrophobicity and isoelectric point (pI) are key physicochemical traits influencing protein localization and functional dynamics within the cell. Typically, hydrophobic proteins associate with membrane compartments, whereas hydrophilic proteins localize in aqueous cellular environments. In addition, a protein’s isoelectric point can affect its intracellular stability and interactions with other molecules. Further analysis revealed that only two proteins from wolfberry exhibited theoretically predicted hydrophobic properties (GRAVY > 0), while the remaining 111 proteins were predicted to be hydrophilic (GRAVY < 0). This in silico analysis suggests that the majority of SL biosynthetic proteins are likely hydrophilic in nature. However, this prediction requires further experimental validation. Additionally, a wide variation was observed in the isoelectric point distribution among the proteins. These findings provide an essential basis for future research on the subcellular localization and biological functions.

### Structural analysis of SL biosynthetic genes

**Fig 2:**
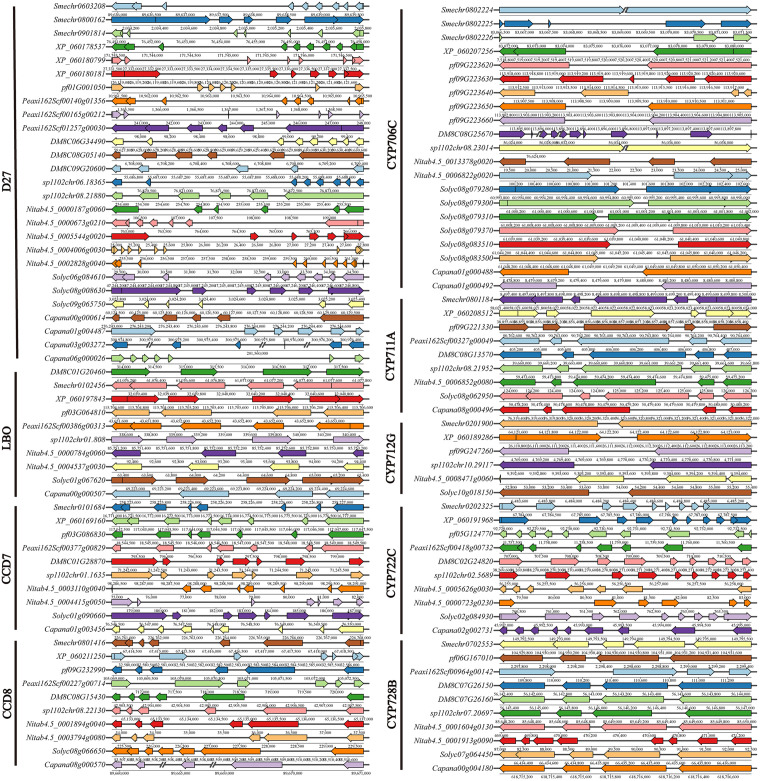
Phylogenetic tree of SL biosynthetic genes from nine Solanaceous species. The gene structures of SL biosynthesis-related genes—including *D27*, *CCD7*, *CCD8*, *LBO*, *CYP711A*, *CYP712G*, *CYP722C*, *CYP728B*, and *CYP706C*—were visualized based on genomic annotations. Each gene model is depicted with colored boxes representing exons, thin lines for introns, and arrows indicating transcriptional direction.

Gene structure analysis revealed that SL biosynthetic genes exhibited extensive variation in exon–intron structure among Solanaceous species Fig 2. Among them, *D27* exhibited the greatest structural variability, with exon numbers ranging from 4 to 7, indicating that this gene family may have undergone structural remodeling during evolution. In contrast, most other gene families maintained highly conserved exon–intron configurations, suggesting a stable structural conservation. Although orthologous gene structures remained relatively stable across species, variations in gene length and intron size were observed, possibly shaped by localized selection or recombination. This study reveals both the conserved and diversified structural evolution of SL biosynthetic genes in Solanaceous species, providing an important basis for understanding their functional differentiation and regulatory mechanisms.

### Prediction of cis-regulatory elements in SL biosynthetic genes

**Fig 3:**
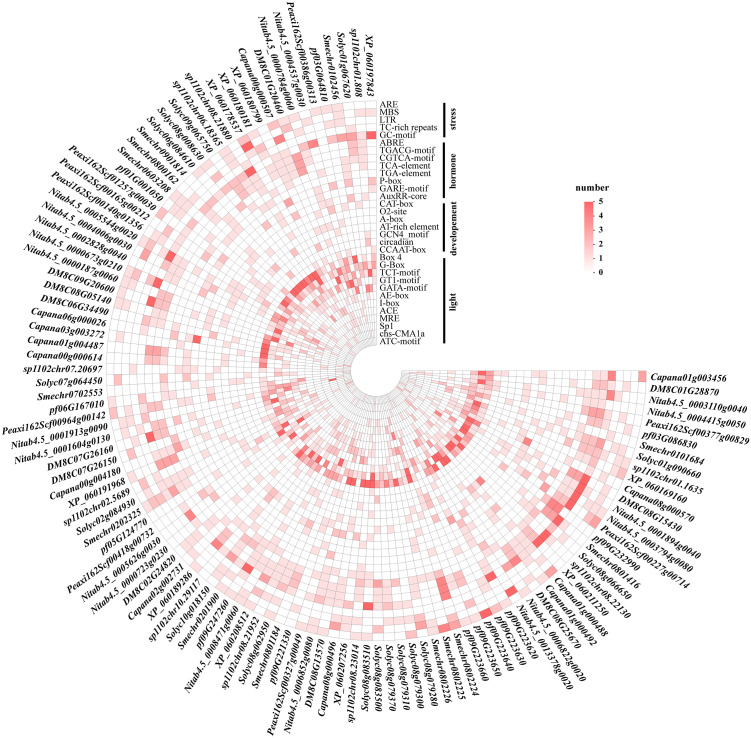
Classification and distribution of cis-regulatory elements in SL biosynthetic gene promoters across different species. Elements were grouped into four functional categories: stress responsiveness, hormone responsiveness, developmental regulation and light responsiveness. The outermost circular axis lists gene IDs from different species, while rows represent individual cis-elements. Color intensity indicates the number of element occurrences, as shown in the legend.

To investigate potential regulatory mechanisms, we analyzed the 2-kb upstream promoter regions of the 113 SL biosynthetic genes identified in nine Solanaceous species Fig 3. A total of 2,500 cis-acting elements were identified and functionally categorized into four classes: light-responsive, phytohormone-responsive, growth and development-related, and abiotic stress responses. Light-responsive elements were the most abundant, accounting for nearly half of the total elements (n = 1,202), and were broadly distributed across most gene promoters. A total of 647 hormone-responsive elements were identified, spanning multiple hormonal pathways including jasmonic acid (JA), salicylic acid (SA), gibberellin (GA), abscisic acid (ABA), and auxin (IAA). Additionally, 406 elements were associated with abiotic stress responses such as low temperature, drought, and hypoxia. Another 245 elements were linked to developmental processes, including circadian control, meristem activity, and endosperm formation. These findings indicate that SL biosynthetic gene promoters are enriched with diverse functional cis-elements, implying that the SL pathway is potentially regulated by hormonal and environmental signals, and their involvement in both stress responses and developmental processes.

### Selection pressure analysis of SL biosynthetic genes in tomato and pepper

To assess the selection pressure acting on SL biosynthetic genes during *Solanaceae* diversification, Ka, Ks and Ka/Ks ratios were calculated for homologous gene pairs between tomato and petunia, as well as pepper and petunia Table 2. The Ka/Ks ratio is commonly used to assess selective pressure on coding genes: Ka/Ks > 1 indicates positive (Darwinian) selection driving functional innovation, Ka/Ks ≈ 1 implies neutral evolution, and Ka/Ks < 1 reflects purifying selection driven by functional constraints to preserve gene integrity. All gene pairs exhibited significantly lower Ka values than Ks, resulting in Ka/Ks ratios consistently below 1. Above all, SL biosynthetic genes across Solanaceous crops exhibited strong purifying selection, purging deleterious alleles while preserving structural and functional integrity of protein. This evolutionary constraint underscores their indispensable roles in mediating critical biological functions in *Solanaceae*.

**Table 2:** Selection pressure analysis of homologous gene pairs.

Homologous Genes	Nonsynonymous Mutation rate(Ka)	Synonymous Mutation Rate(Ks)	The Ratio of Nonsynonymous Mutation Rate to Synonymous Mutation Rate(Ka/Ks)
*Peaxi162Scf00140g01356-Solyc06g084610*	0.10	0.48	0.21
*Peaxi162Scf00165g00212-Solyc09g065750*	0.11	0.55	0.20
*Peaxi162Scf00227g00714-Solyc08g066650*	0.06	0.69	0.08
*Peaxi162Scf00327g00049-Solyc08g062950*	0.07	0.40	0.17
*Peaxi162Scf00377g00829-Solyc01g090660*	0.07	0.63	0.11
*Peaxi162Scf00386g00313-Solyc01g067620*	0.04	0.39	0.09
*Peaxi162Scf00418g00732-Solyc02g084930*	0.06	0.44	0.15
*Peaxi162Scf00964g00142-Solyc07g064450*	0.12	0.49	0.24
*Peaxi162Scf01257g00030-Solyc08g008630*	0.12	0.82	0.15
*Peaxi162Scf00140g01356-Capana06g000026*	0.13	0.36	0.36
*Peaxi162Scf00165g00212-Capana03g003272*	0.11	0.42	0.26
*Peaxi162Scf00227g00714-Capana08g000570*	0.06	0.53	0.12
*Peaxi162Scf00327g00049-Capana08g000496*	0.06	0.30	0.19
*Peaxi162Scf00377g00829-Capana01g003456*	0.07	0.50	0.14
*Peaxi162Scf00386g00313-Capana00g000507*	0.03	0.37	0.09
*Peaxi162Scf00418g00732-Capana02g002731*	0.07	0.34	0.21
*Peaxi162Scf00964g00142-Capana00g004180*	0.11	0.54	0.21
*Peaxi162Scf01257g00030-Capana00g000614*	0.08	0.53	0.15
*Peaxi162Scf01257g00030-Capana01g004487*	0.08	0.53	0.15

### Tertiary structure and subcellular localization prediction of SL biosynthetic proteins in tomato and pepper

**Fig 4:**
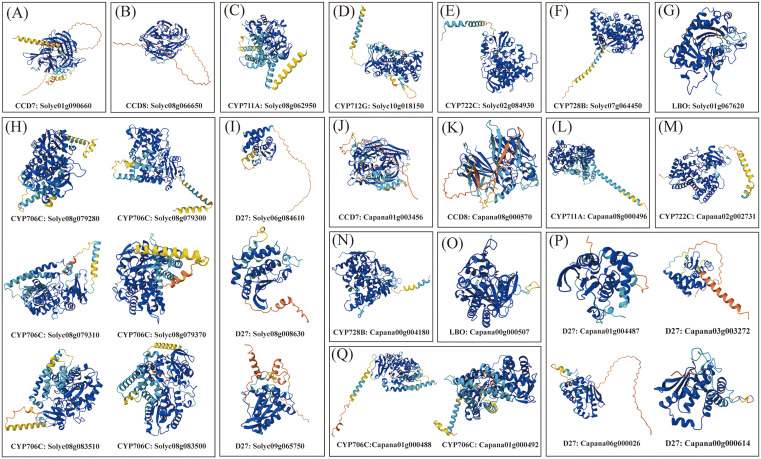
Predicted 3D protein structures of SL biosynthetic genes in tomato and pepper. This figure presents the AlphaFold-predicted 3D structures of proteins encoded by SL biosynthesis-related genes from *Solanum lycopersicum* (Solyc) and *Capsicum annuum* (Capana). Panels A–H display tomato proteins, and panels I–Q show corresponding proteins from pepper.

Secondary structure analysis revealed that SL biosynthetic proteins in tomato and pepper primarily consisted of α-helices, extended strands, and random coils, with β-turn motifs absent from all sequences S4 Table. D27, CCD7, CCD8, and LBO proteins were enriched in random coil content (51.52%–69.08%), while α-helices and extended strands accounted for 11.13%–39.09% and 6.9%–22.26%, respectively. In contrast, CYP711A, CYP722C, CYP712G, CYP728B, and CYP706C proteins display enriched α-helical (43.58%–44.27%) and random coil (46.84%–50.47%) content, with reduced extended strand representation (8.89%–11.52%). Further analysis of 3D structural modeling revealed remarkable conservation among six tomato CYP706C proteins, likely due to tandem duplications that have produced highly identical amino acid sequences. This structural conservation could suggest potential redundancy or coordinated regulation of their biochemical functions. Subcellular localization prediction suggested that most SL biosynthetic proteins are predicted to localize to plastids or the cytoplasm, which is consistent with their potential involvement in the methylerythritol Pi (MEP) pathway or cytoplasmic metabolic processes. Interestingly, species-specific localization was predicted for certain proteins: CCD7 in pepper, as well as D27 and CYP712G in tomato, were predicted to be nuclear-localized, suggesting possible functions in signal perception or transcriptional regulation within the nucleus. In summary, the observed variations in secondary structure elements, three-dimensional configurations, and predicted subcellular localization of SLs biosynthetic proteins between tomato and pepper reflect both evolutionary conservation and diversification arising from gene duplication and species-specific differentiation Fig 4. These structural and spatial differences may underlie functional divergences, such as substrate preference, enzymatic regulation, and signaling mechanisms.

### Expression analysis of SL biosynthetic genes in tomato and pepper under Pi deficiency

**Fig 5:**
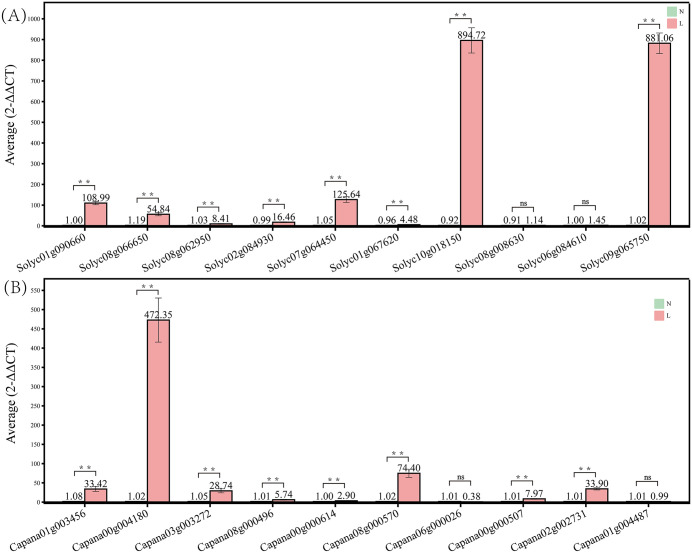
Relative expression levels of SL biosynthetic genes under normal (N) and Pi-deficient (L) conditions in tomato (A) and pepper (B). Gene expression levels were quantified by qRT-PCR and calculated using the 2^^-ΔΔCT^ method. ** significant differences between treatments at p < 0.01; ns: no significant difference. Data represent the mean ± SD for three biological replicates (n = 3).

Previous studies have shown that Pi deficiency triggers transcriptional activation of SL biosynthetic genes in various plant species. To assess whether a similar regulatory mechanism exists in Solanaceous crops with contrasting *Orobanche* susceptibility, we analyzed the expression of SL biosynthetic genes in the roots of tomato (susceptible) and pepper (resistant) under Pi-deficient conditions Fig 5 and S5 Table. Most genes were significantly upregulated under Pi-deficient conditions. Notably, members of the D27 gene family—including *Solyc06g084610* and *Solyc08g008630* in tomato and *Capana01g004487* and *Capana06g000026* in pepper—did not exhibit differential expression between Pi-replete and Pi-deficient conditions. These results suggest that these specific gene members may either be unresponsive to Pi signaling or may not be involved in SL biosynthesis pathways.

## Discussion

In this study, we systematically identified 10 gene families in the SL biosynthesis pathway and obtained 113 genes across nine Solanaceous species. However, their distribution varied significantly among species, suggesting gene expansion or loss during *Solanaceae* diversification. Across all surveyed species, genes involved in the upstream synthesis of CLA were highly conserved, whereas the downstream CYP450 family genes associated with canonical and non-canonical SL biosynthesis exhibited pronounced species-specificity, consistent with observations in rice and sorghum [9,30]. These findings suggest that while the core SL biosynthetic pathway is evolutionarily stable, the diversification of downstream components may reflect adaptive functional divergence shaped by distinct ecological or physiological requirements. Previous studies have shown that knocking out/down key genes in the CLA biosynthetic pathway (such as *CCD7*, *CCD8*, and *CYP711A*) in tomato and other species significantly reduces *Orobanche* seed germination and parasitism, while also causes excessive shoot branching [24–27]. However, the canonical SLs are not essential for shoot branching regulation. For instance, blocking the biosynthesis of canonical SLs in rice can effectively suppress Striga parasitism without affecting host plant growth, and *CYP722C* knockout in tomato similarly reduces *Orobanche* seed germination without affecting branching [7,25–27,38]. By contrast, LBO, a key enzyme for non-canonical SL biosynthesis, is highly conserved in tomato, maize, and other crops, and has been implicated in the regulation of shoot branching, although its role in parasitic seed germination remains to be elucidated [28,30,39]. Together, current evidence suggests that canonical SLs regulate *Orobanche* germination but not shoot branching, whereas 1′-OH-MeCLA, synthesized by LBO, likely functions as the shoot branching hormone [28].

In this study, CYP728B was identified in pepper, whereas both CYP712G and CYP728B were found in tomato. This gene distribution pattern raises the possibility that tomato may synthesize a more diverse blend of canonical SLs, while pepper might produce a comparatively simpler profile. We hypothesize that this difference in the potential repertoire of SL structures could be a contributing factor to the observed differential *Orobanche* germination rates between these two species, as previous studies have shown that mixtures of canonical SLs with different C-ring structures can reduce germination activity [40–42]. However, this model requires direct experimental validation. Regarding the significant difference in parasitism rates between pepper and tomato, further investigation of the host–parasite interaction during the invasion process is needed to provide additional mechanistic insight [22]. It is also worth noting that, while LBO and CYP706C were identified across all nine *Solanaceae* species, the CLAMT protein, a key enzyme in non-canonical SL biosynthesis, was not identified. The consistent absence of CLAMT across all nine Solanaceae species leads us to propose a hypothesis that these plants may have evolved alternative biosynthetic pathways for non-canonical SLs, and testing this hypothesis should be a priority for future research. Whether there are unidentified functionally redundant enzymes, or whether species-specific evolutionary divergence exists in the biosynthesis of non-canonical SLs, remains to be further investigated.

Through analysis of cis-regulatory elements in promoter regions, this study revealed that many SL biosynthetic genes contain elements associated with abiotic stress responses, including low temperature, drought, and hypoxia, as well as hormone-responsive elements linked to multiple phytohormones such as IAA, GA, ABA, JA, and SA. This suggests that SL biosynthesis may participate in the regulation of plant stress responses through a complex signaling network involving other hormones. Previous reports have shown that SLs can cooperate with ABA and IAA to modulate root system architecture and improve stress adaptation [2,43,44]. The prevalence of stress- and hormone-responsive cis-elements in SL biosynthetic gene promoters provides a compelling basis for hypothesizing that these genes are integrated into complex signaling networks that respond to both abiotic and biotic cues. Future work should aim to experimentally dissect these proposed regulatory relationships under controlled stress conditions.

In addition, we analyzed the expression levels of key SL biosynthetic genes in tomato and pepper under Pi-deficient conditions. The results showed that most of these genes were significantly upregulated under low-Pi stress, further confirming that Pi deficiency can promote SL synthesis and increase SL content in the rhizosphere. These findings, consistent with established literature [18], provide a molecular rationale for the observed agronomic phenomenon where nutrient management can influence parasitic weed infestation. Our data, showing that Pi deficiency upregulates SL biosynthetic genes, strengthen the argument that managing soil nutrient levels, particularly phosphorus, could be a promising approach to modulate rhizosphere SL levels and potentially mitigate *Orobanche* risk. Future field trials are warranted to directly test the efficacy of precision fertilizer application as a sustainable management strategy. Overall, *Orobanche* remains a major global threat to crop production, and the molecular mechanisms of its host recognition and parasitism are still not fully understood [14]. Future research should integrate multi-omics datasets, regulatory network analyses, and functional experiments to construct a comprehensive regulatory map of SL biosynthesis, uncover the dynamic regulatory mechanisms under complex stress conditions, and provide theoretical and technical support for breeding parasite-resistant, stress-resilient, and high-yield Solanaceous crops.

Future research on the evolution and regulatory mechanisms of SL biosynthesis in Solanaceous species must address several critical gaps. Firstly, the functional boundaries between canonical and non-canonical SLs in mediating parasitic plant recognition and regulating host development remain undefined. Comprehensive studies using heterologous expression systems, metabolite profiling, and functional mutant characterization are essential to clarify the biosynthetic gene functions and mechanisms of distinct SL classes. Secondly, the biosynthetic pathway for non-canonical SLs remains poorly understood, particularly regarding the functional replacement of missing key enzymes such as CLAMT. This will require further investigation using transcriptomics, protein interaction networks, and metabolomics. Furthermore, the regulatory networks involving SLs in response to multiple abiotic stresses, and their synergistic interactions with other plant hormones, also remain largely unexplored. Advanced approaches such as single-cell transcriptome analysis, dynamic hormone signaling monitoring, and precision genome editing to decode SL-mediated dynamic regulation and network integration mechanisms. Ultimately, constructing an integrated model linking genes, regulatory elements, hormones, metabolites, and phenotypes will lay the foundation for breeding Solanaceous crops with broad-spectrum resistance and improved environmental adaptability.

It is important to note that the findings and evolutionary interpretations presented in this study are derived from a comprehensive bioinformatic analysis. While we have employed stringent criteria for gene identification and analysis, the functional implications and causal relationships proposed herein remain as robust, testable hypotheses. Furthermore, while the selection of tomato and pepper for expression analysis provided a strategically focused comparison between susceptible and resistant phenotypes, it necessarily limited the scope of our experimental validation. Future studies encompassing a broader phylogenetic range of Solanaceous species will be essential to fully elucidate the diversity of SL gene regulation and its role in shaping host-parasite interactions.

## Conclusions

Overall, this in silico study provides a comprehensive genome-wide analysis of potential SL biosynthetic genes across nine Solanaceous species, revealing both conserved and species-specificity in gene distribution, structure, and evolution. While upstream CLA biosynthetic genes are highly conserved, downstream CYP450-related genes show substantial species-specific divergence, potentially reflecting ecological adaptation. Collectively, these computational findings generate testable hypotheses about SL pathway evolution in Solanaceae and identify candidate genetic elements for future functional studies aimed at improving crop resistance traits.

## Supporting information

S1 Table Summary of previously reported SL biosynthetic protein sequences from the literature. (XLSX)

S2 Table List of gene-specific primers used for qRT-PCR in this study. (XLSX)

S3 Table Basic information of SL biosynthetic proteins identified in nine Solanaceous genomes. (XLSX)

S4 Table Structural composition and localization predictions for SL biosynthesis-related proteins in Solanum lycopersicum and Capsicum annuum. (XLSX)

S5 Table The qRT-PCR results of SL biosynthetic genes under Pi-deficient and normal conditions. (XLSX)
